# Prevalence and associated risk factors for anxiety and depression in infertile couples of ART treatment: a cross-sectional study

**DOI:** 10.1186/s12888-022-04256-9

**Published:** 2022-09-19

**Authors:** Li Zhang, Hongfang Shao, Mian Huo, Jie Chen, Minfang Tao, Zhangshun Liu

**Affiliations:** Department of Reproductive Medicine Center, Shanghai Jiao Tong University Affiliated Sixth People’s Hospital, Shanghai, 200233 People’s Republic of China

**Keywords:** Anxiety, Depression; infertile couple, Risk factors

## Abstract

**Background:**

Infertility now is a public health concern and is associated with increased psychological distress.

**Methods:**

We enrolled 1247 infertile couples and assessed their anxiety and depression status before and during assisted reproductive technology (ART) treatment using the Self-Rating Anxiety Scale (SAS) and Self-Rating Depression Scale (SDS). The Chi-square or fisher’s exact test was used to analyze the prevalence of anxiety and depression in infertile couples. Multivariate logistical regression was performed to analyze the risk factors for anxiety and depression.

**Results:**

The prevalence of anxiety was 13.5% and 8.7% (*p* < 0.05), and that of depression was 9.4% and 7.9% (*p* = 0.2) in female and male partners, respectively. Female SAS and SDS scores were positively associated with male SAS and SDS scores, respectively (*r* = 0.52 and *r* = 0.50, respectively, both *p* < 0.0001), and were positively associated with their own SDS and SAS scores, respectively (*r* = 0.63 and *r* = 0.62, respectively, both *p* < 0.0001). Their own depression or partners’ anxiety was associated with the anxiety, and their own anxiety or partners’ depression was associated with the depression in infertile couples. No children, unemployment, and low education level were also associated with female anxiety. SAS and SDS scores were significantly decreased during ART treatment.

**Conclusions:**

Females were more vulnerable to having anxiety than males in infertile couples. Anxiety and depression in infertile couples could interact, therefore, anxiety and depression would be simultaneously counseled, and their partners also should be given supportive psychotherapy.

**Trial registration:**

It was an observational study and had no health care interventions on participants. So it was not registrated.

## Background

The inability to conceive after at least 12 months of regular, unprotected sexual intercourse was defined as infertility [1]. The number of infertility was rising globally over the last several decades, with an annual percentage increase of 0.370% in women and 0.291% in men from 1990 to 2017 [2], and reached 8–12% in reproductive couples [3]. Infertility is one of the most stressful events that the pressure from traditional ideals, families, and society can make adverse psychological and mental impacts including loss of self-esteem, lack of self-confidence, and even psychiatric disorders for infertile couples [4]. It was estimated that the prevalence of anxiety and depression in infertile women reached 23.2% and 17% in China, respectively [5]. Therefore, infertility is not only a medical problem but also a public health issue.

Assisted reproductive technology (ART) treatment was an effective solution to infertility that was widely accepted by infertile couples. However, ART is costly and needs a series of complex processes such as ovarian stimulation, retrieving oocytes, and transferring embryos into the woman’s uterus [6], which can increase the psychological burden on infertile couples, especially on the female partner [7]. The success rate of in vitro fertilization (IVF) is about 30% per cycle [8], so most infertile couples would face failure. Some women dropped out of treatment [9] or even never started the treatments [10] due to these reasons. More importantly, the psychological state of infertile couples could affect the outcome of treatment. Women with higher stress and anxiety scores on the day prior to oocyte retrieval had a lower probability of pregnancy [11]. There were significant associations between depression and anxiety scores before ART treatment and reduced pregnancy chances with ART [12, 13]. Therefore, it needs a health mental state to face ART treatment, and psychosocial intervention is of crucial importance for infertile couples.

To offer effective counseling and psychological support, it is necessary to fully understand the risk factors for adverse psychology especially anxiety and depression in infertile couples. A previous study showed that anxiety and depression scores were inversely correlated with their ages in women and were significantly correlated with the duration of infertility in men [14]. In infertile couples, the incidence of anxiety in women was related to age, education level, and family income, and the incidence of depression was related to the age and duration of infertility [15]. However, most previous studies mainly enrolled infertile women while men were underrepresented [16, 17]. In addition, less attention has been paid to the interaction between psychological disorders, and between female and male partners. Here, we enrolled 1247 infertile couples undergoing ART and conducted a longitudinal observational study with the purpose of evaluating the prevalence of depression and anxiety, and comprehensively analyzing the risk factors, especially the association between anxiety and depression in infertile couples.

## Methods

### Subjects and ethics statement

Infertile couples who planned to undergo ART at the Centre of Reproductive Medicine of Shanghai Jiao Tong University Affiliated Sixth People's Hospital between January 2016 and December 2018 were consecutively recruited in our study. The inclusion criteria were as follows: infertile couples could complete the ART treatment and the Self-Rating Anxiety Scale (SAS) and Self-Rating Depression Scale (SDS) survey before and during ART treatment. The exclusion criteria included subjects who had a history of mental illness or psychiatric disorders; the presence of complications during the ART cycle (such as ovarian hyperstimulation syndrome, oocyte retrieval bleeding, pelvic infection, etc.). All subjects did not undergo psychological counseling before enrollment. The study was approved by the Institutional Review Board of Shanghai Jiao Tong University Affiliated Sixth People's Hospital. All methods were carried out in accordance with relevant guidelines and regulations. Written informed consent was provided by all subjects.

### Questionnaires and data collection

All subjects completed two questionnaires: SDS, designed by Zung in 1965 [18], and SAS, designed by Zung in 1971 [19] at the following stages: (i) on the first day of ovarian stimulation (Visit 1), (ii) on the day of oocyte retrieval (Visit 2), and (iii) on the day of embryo transfer (Visit 3). SAS and SDS questionnaires have been validated and are widely used for assessing an individual’s mental state [15–17]. Both SAS and SDS questionnaires cover 20 questions. Each question is scored on a 4-point scale ranging from 1 (none, or a little of the time) to 4 (most, or all of the time). The raw total scores are obtained by summarizing the scores of the 20 questions and are converted to percentile standard scores. The subjects with SAS scores ≥ 50 are diagnosed with anxiety, while scores ranging from 50 to 59 are classified as “mild”, from 60 to 69 are “moderate”, and more than 69 are “severe” [15]. The subjects with SDS scores ≥ 53 are identified as depression, while scores ranging from 53 to 62 are “mild”, from 63 to 72 are “moderate”, and more than 72 are “severe” [15].

A researcher formally interviewed face to face all enrolled subjects. Sociodemographic data (such as age, education, and profession), and clinical data including reproductive history, previous treatments, and the causes of infertility were collected from ART medical records.

### Statistical analysis

We analyzed all data using SPSS software, version 24.0 (SPSS Inc., Chicago, IL, USA). For categorical variables, we calculated proportions. For continuous variables, we calculated the mean and standard deviation. We compared categorical variables, such as the prevalence of anxiety and depression in infertile couples, using the Chi-square test or Fisher’s exact test. We used the repeated measures ANOVA test (including Tukey post hoc correction) to compare the SAS and SDS scores of infertility couples on Visit 1, Visit 2, and Visit 3. Factors between anxiety and no anxiety (or between depression and no depression) were firstly analyzed by univariate analysis, when factors with a *p* < 0.1 were included in the multivariate logistical regression. We considered *p* < 0.05 at two-sided to be statistically significant.

## Results

### Clinical characteristics of the enrolled infertile couples

**Fig. 1:**
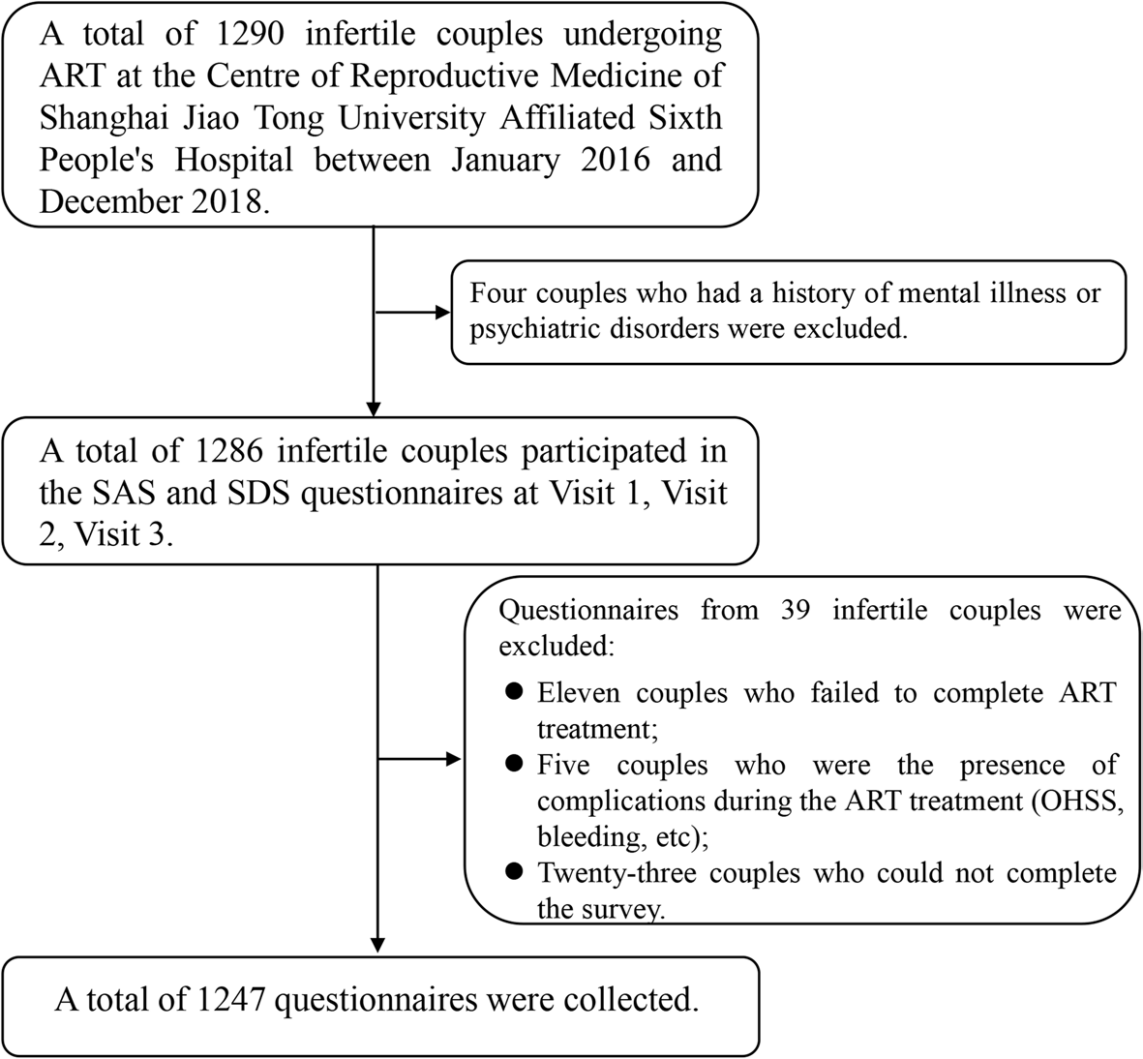
Flowchart of participant enrollment and study design. SAS, Self-Rating Anxiety Scale; SDS, Self-Rating Depression Scale; ART, assisted reproductive technology; OHSS, ovarian hyperstimulation syndrome; Visit 1, the initiation day; Visit 2, the oocyte retrieval day; Visit 3, the embryo transplantation day

As shown in Fig. 1, there were 1290 infertile couples undergoing ART in our center between January 2016 and December 2018. Four couples were excluded because they had a history of mental illness or psychiatric disorders. In addition, 11 couples who failed to complete ART treatment, 5 couples who were present with complications during the ART treatment, and 23 couples who could not complete the survey, were excluded. Therefore, a total of 1247 infertile couples were enrolled in this study. The clinical characteristics of the 1247 infertile couples were shown in Table 1. The age was 31.64 ± 5.11 years for female partners and 33.33 ± 5.88 years for male partners. The college degree was dominant in infertile couples and accounted for 54.5%(679/1247) and 55.6% (693/1247) in males and females, respectively. The majority of male partners were employed (96.8%,1207/1247), but 249 (20%) of female partners were unemployed. The duration of infertility was 3.55 ± 2.59 years. The causes of infertility were 40.3%(503/1247) for female factors, 20.3%(253/1247) for male factors, 36.3% (453/1247) for both female and male factors, and 3.0%(38/1247) for unknown factors.

**Table 1:** The clinical characteristics of 1247 infertile couples

**Variables**	
**Age(years)**	
Female	31.64 ± 5.11
Male	33.33 ± 5.88
**Causes of infertility, n(%)**	
Male factors	253(20.3)
Female factors	503(40.3)
Both factors	453(36.3)
Unknown	38(3.0)
**Duration of infertility,(years)**	3.55 ± 2.59
**Employment status, n(%)**	
**Male**	
Yes	1207(96.8)
No	40(3.2)
**Female**	
Yes	998(80)
No	249(20)
**Education level, n(%)**	
**Male**	
Junior high school and lower	233(18.7)
High school	238(19.1)
College degree	679(54.5)
Above college degree	97(7.8)
**Female**	
Junior high school and lower	287(23)
High school	192(15.4)
College degree	693(55.6)
Above college degree	75(6.0)

### Female was more likely to have anxiety than male in infertile couples

To analyze the psychological state of infertile couples, the anxiety and depression of 1247 infertile couples planning ART were assessed using SAS and SDS. As shown in Table 2, the prevalence of anxiety in female partners was 13.5% (168/1247) and was higher than that in male partners [8.7% (108/1247)] (*p* < 0.05). Of them,148 females (88.1%,148/168) were mild anxiety, and 20 females (11.9%, 20/168) were moderate anxiety (*p* < 0.05). Mild, moderate, and severe anxiety accounted for 76.9% (83/108), 21.3% (23/108), and1.9% (2/108) among 108 male partners with anxiety, respectively. The number of mild anxiety was significantly more than those of moderate, and severe anxiety (both *p* < *0.05*). The prevalence of depression was 9.4%(117/1247) and 7.9%(99/1247) in female and male partners, respectively (*p* = *0.2*). Similar to anxiety, most of them were mild depression, which was 75.2% (88/117) and 78.8% (78/99) in female and male partners, respectively, and were higher than moderate, and severe depression (all *p* < *0.05*).

**Table 2:** The prevalence of anxiety and depression in 1247 infertile couples

	**Anxiety**		**Depression**	
Variables	Female	Male	Female	Male
Prevalence,%(n/N)	13.5 (168/1247)	8.7 (108/1247)^a^	9.4 (117/1247)	7.9 (99/1247)
**Degree**				
Mild,%(n/N)	88.1 (148/168)^b^	76.9 (83/108)^c^	75.2 (88/117)^d^	78.8 (78/99)^e^
Moderate,%(n/N)	11.9 (20/168)	21.3 (23/108)	22.2 (26/117)	21.2 (21/99)
Severe,%(n/N)	0 (0/168)	1.9 (2/108)	2.6 (3/117)	0 (0/99)

Data are presented as % (n/N), “n” refers to the number of cases, and “N” refers to the total number. ^a^There was a significant difference between the prevalence of anxiety in females and that in males (*p* < 0.05); ^b^There were significant differences between the percentage of mild anxiety and the percentage of moderate anxiety or severe anxiety in females (*p* < 0.05); ^c^There were significant differences between the percentage of mild anxiety and the percentage of moderate anxiety or severe anxiety in males (*p* < 0.05); ^d^There were significant differences between the percentage of mild depression and the percentage of moderate depression or severe depression in females (*p* < 0.05); ^b^There were significant differences between the percentage of mild depression and the percentage of moderate depression or severe depression in males (*p* < 0.05)

**Fig. 2:**
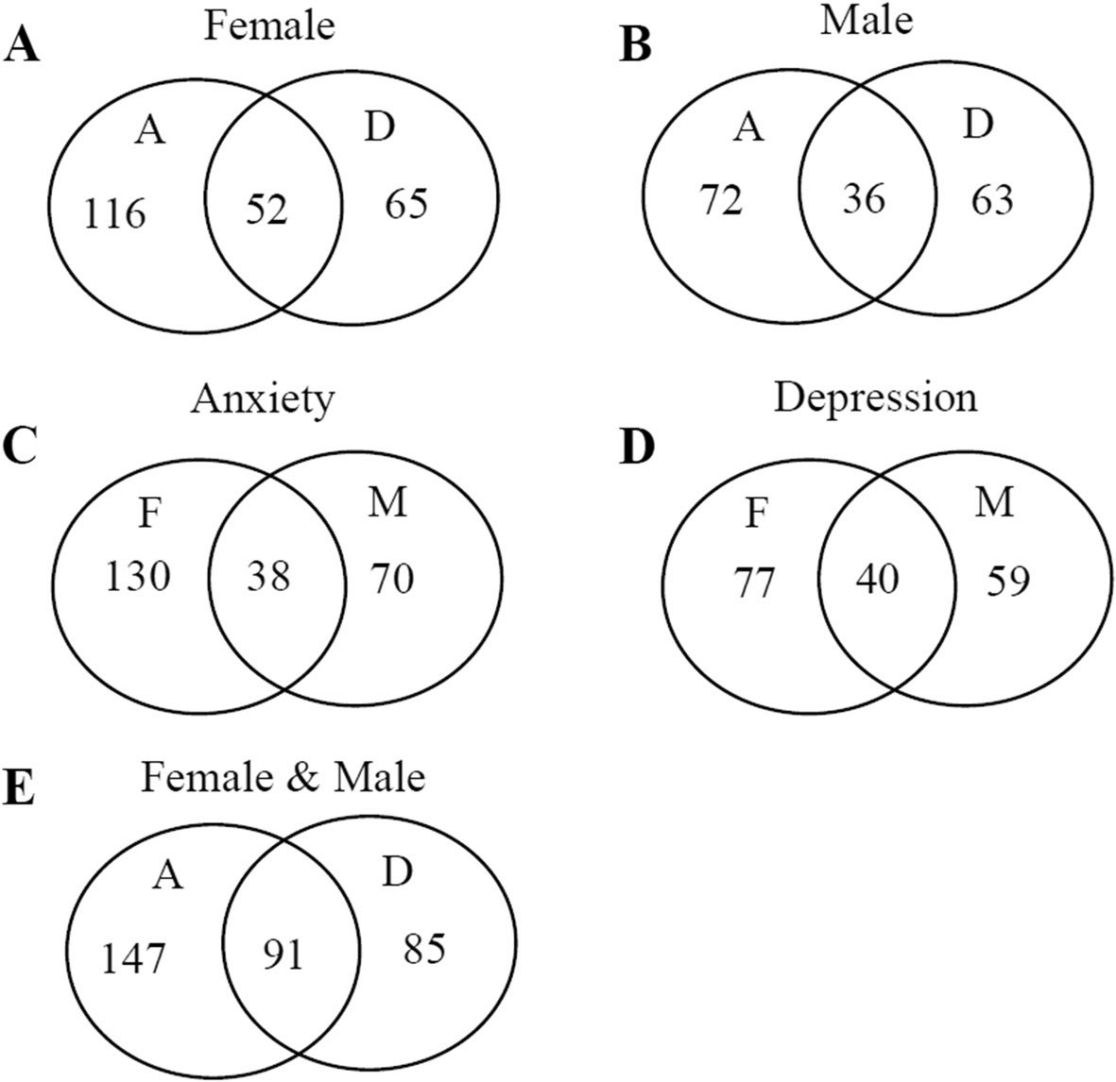
The prevalence of anxiety and depression in 1247 infertile couples. **A** The prevalence of anxiety and depression in female partners; **B** The prevalence of anxiety and depression in male partners; (**C**)The prevalence of anxiety in 1247 infertile couples; (**D**)The prevalence of depression in 1247 infertile couples; (**E**)The prevalence of anxiety and depression in 1247 infertile couples. A, anxiety; D, depression; F, female; M, male

Among 1247 infertile couples, 233 females (18.7%, 233/1247) had anxiety and/or depression, of whom 52 females (22.3%,52/233) were comorbidity of anxiety and depression (Fig. 2A). A total of 171 males (13.7%, 171/1247) had anxiety and/or depression, of whom 36 males (21.1%, 36/171) were comorbidity of anxiety and depression (Fig. 2B). The prevalence of anxiety and/or depression in females was higher than that in males (18.7% vs 13.7%, *p* = *0.0009*). 238 infertile couples with one or both partners (19.1%,238/1247) had anxiety. Of them, 38 infertile couples (16.0%,38/238) had anxiety in both partners (Fig. 2C). 176 infertile couples with one or both partners (14.1%,176/1247) had depression. Of them, 40 infertile couples (22.7%,40/176) had depression in both partners (Fig. 2D). The prevalence of anxiety was higher than depression in infertile couples (19.1% vs 14.1%, *p* = *0.0008*). A total of 323 infertile couples (25.9%,323/1247) with at least one partner had anxiety, depression, or both (Fig. 2E).

### The levels of anxiety and depression were positively associated between female and male partners in infertile couples

**Fig. 3:**
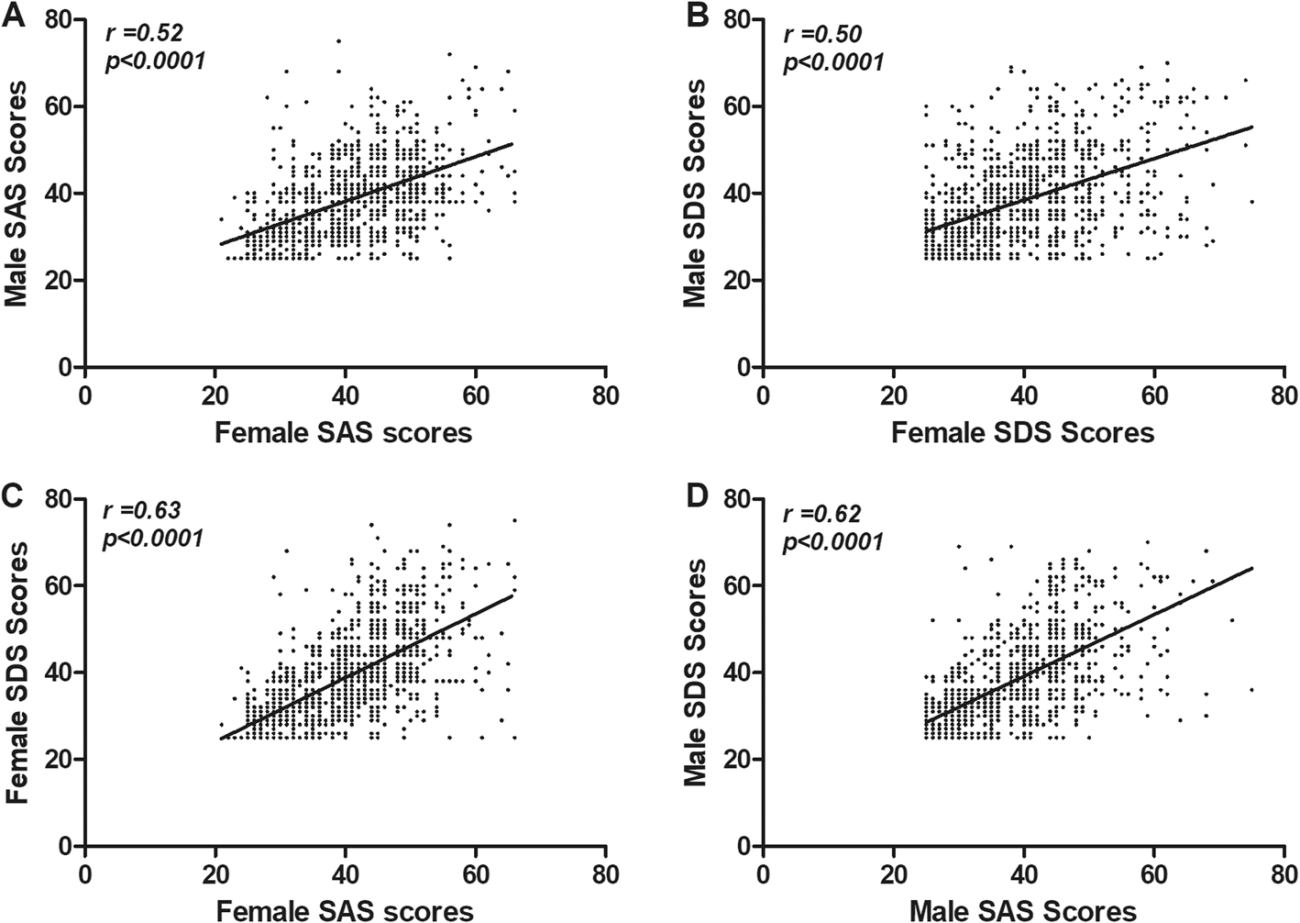
The Correlation of SAS and SDS scores before treatment. **A** The correlation of SAS scores between female and male partners; (**B**) The correlation of SDS scores between female and male partners; (**C**) The correlation between female SDS scores and female SAS scores; (**D**) The correlation between male SDS scores and male SAS scores

To investigate the interaction of different psychological disorders in infertile couples, we analyzed the association of SAS and SDS scores between female and male partners. As shown in Fig. 3A&B, female SAS and SDS scores were both positively associated with male SAS and SDS scores (*r* = 0.52 and *r* = 0.50, both *p* < *0.0001*, respectively). In addition, female SAS scores were correlated with their own SDS scores (*r* = 0.63, *p* < *0.0001*) (Fig. 3C). Male SAS scores were correlated with their own SDS scores (*r* = 0.62, *p* < *0.0001*) (Fig. 3D).

### Their own or partners’ anxiety or depression were risk factors for anxiety and depression in infertile couples

To further discover the risk factors for anxiety and depression in infertile couples, we analyzed the association between the education levels, age, the causes of infertility, no children, their partners’ anxiety/depression, their own anxiety/depression, duration of disease, the state of employment, and the anxiety/depression using multivariate logistic regression. As shown in Table 3, no children (odds ratio (OR) 2.12, *p* = *0.04*), their own depression (OR 6.03, *p* < *0.0001*), their partners’ anxiety (OR 3.45, *p* < *0.0001*), unemployment (OR 1.69, *p* = *0.02*) were associated with female anxiety. In addition, compared to the above college degree, a high school degree (OR 5.49, *p* = *0.04*), and a college degree (OR 4.84, *p* = *0.04*) were also risk factors for female anxiety. Their own depression (OR 3.42, *p* < *0.0001*) and their partners’ anxiety (OR 6.94, *p* < *0.0001*) were positively associated with male anxiety. Their own anxiety (OR 5.88, *p* < *0.0001*) and their partners’ depression (OR 6.67, *p* < *0.0001*) were positively associated with female depression. Similarly, their own anxiety (OR 7.14, *p* < *0.0001*) and their partners’ depression (OR 6.67, *p* < *0.0001*), were positively associated with male depression.

**Table 3:** Multivariate analysis of the risk factors associated with anxiety and depression of infertile couples

Variables	Female anxiety			Male anxiety			Female depression			Male depression		
	**Adjusted OR**	**95% CI**	***p-value***	**Adjusted OR**	**95% CI**	***p-value***	**Adjusted OR**	**95% CI**	***p-value***	**Adjusted OR**	**95% CI**	***p-value***
**Age,years(male)**	0.98	0.93–1.03	0.32	*	*	*	1.01	0.95–1.07	0.78	*	*	*
**Age,years(female)**	1.01	0.96–1.07	0.67	*	*	*	0.95	0.89–1.01	0.11	*	*	*
**Education levels(female)**									0.17	*	*	*
Junior high school and lower	4.14	0.83–20.63	0.08	2.26	0.62–0.17	0.47	1.18	0.28–4.93	0.82			
high school	5.49	1.13–26.70	0.04	1.87	0.51–0.14	0.31	0.78	0.19–3.31	0.74			
college degree	4.84	1.09–21.55	0.04	2.43	0.79–0.26	0.68	0.55	0.15–2.01	0.36			
above college degree	1.00			1.00			1.00					
**Education levels(male)**												
Junior high school and lower	0.75	0.28–2.00	0.57	10.20	2.77–0.75	0.13	4.28	0.81–22.67	0.09	6.58	0.69–62.74	0.10
high school	0.77	0.31–1.95	0.59	9.67	2.80–0.81	0.10	1.95	0.37–10.29	0.43	5.46	0.58–51.08	0.14
college degree	0.67	0.30–1.48	0.32	4.85	1.58–0.52	0.42	3.21	0.70–14.74	0.13	2.99	0.34–26.19	0.32
above college degree	1.00						1.00			1.00		
**The causes of infertility**	*	*	*	*	*	*				*	*	*
Female							1.97	0.43–9.04	0.38			
Male							0.89	0.18–4.35	0.88			
Both							1.67	0.36–7.75	0.51			
Unknown							1.00					
**Duration of disease**	*	*	*	*	*	*	*	*	*	*	*	*
**no children**	2.12	1.05–4.25	0.04	*	*	*	*	*	*	*	*	*
**anxiety**							5.88	3.57–9.09	< 0.0001	7.14	4.17–12.50	< 0.0001
**depression**	6.03	3.79–9.59	< 0.0001	6.94	4.03–11.9	< 0.0001						
**the partner anxiety**	3.45	2.11–5.63	< 0.0001	3.42	2.09–5.59	< 0.0001	1.47	0.74–2.93	0.28	1.41	0.81–2.44	0.22
**the partner depression**	1.46	0.85–2.52	0.17	2.86	1.46	0.27	6.67	3.85–12.5	< 0.0001	6.67	3.85–11.11	< 0.0001
**Unemployment(Female)**	1.69	1.11–2.57	0.02	*	*	*	*	*	*	*	*	*
**Unemployment(Male)**	*	*	*	*	*	*	*	*	*	*	*	*

*OR* Odds ratio

^*^Excluded in the multivariate logistic regression model

### The SAS and SDS scores of infertility couples during ART significantly declined

**Fig. 4:**
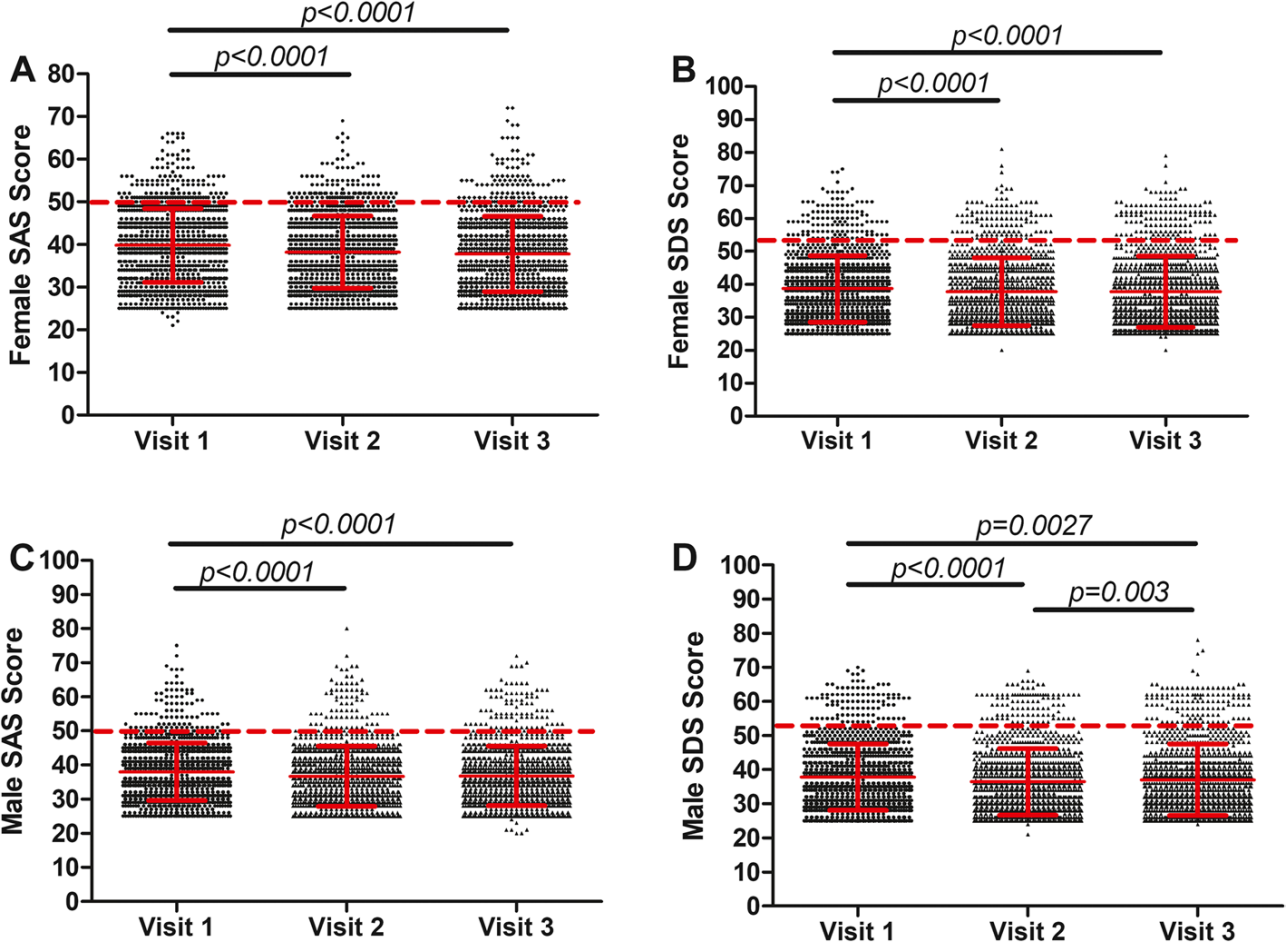
The SAS and SDS scores of infertility couples during the ART treatment. (**A**) Female SAS scores of 1247 infertility couples on Visit 1, Visit 2 and Visit 3; (**B**) Female SDS scores of 1247 infertility couples on Visit 1, Visit 2 and Visit 3; (**C**) Male SAS scores of 1247 infertility couples on Visit 1, Visit 2 and Visit 3; (**D**) Male SDS scores of 1247 infertility couples on Visit 1, Visit 2 and Visit 3. Visit 1, the initiation day; Visit 2, the oocyte retrieval day; Visit 3, the embryo transplantation day. The red lines showed as mean ± SD; The red dotted lines indicated that the SAS score was 50 and the SDS score was 53, respectively

To investigate the dynamic change of psychological states undergoing ART, we compared the SAS and SDS scores of 1247 infertility couples on the initiation day (Visit 1), oocyte retrieval day (Visit 2), and embryo transplantation day (Visit 3). As shown in Fig. 4A, female SAS scores were 39.81 ± 8.62,38.22 ± 8.46, and 37.79 ± 8.83 on Visit 1, Visit 2, and Visit 3, respectively, which significantly decreased compared to Visit 1(both *p* < *0.0001*). Female SDS scores were 38.67 ± 10.04, 37.78 ± 10.28, and 37.78 ± 10.83 on Visit 1, Visit 2 and Visit 3, respectively. The scores on Visit 2 and Visit 3 were lower than that on Visit 1(both *p* < *0.0001*) **(**Fig. 4B**)**. Similar to females, male SAS and SDS scores on Visit 2 and Visit 3 were all lower than those on Visit 1(all *p* < *0.0001*) (Fig. 4C&D).

## Discussion

Infertility now is a public health concern affecting millions of couples [20]. It has an immense impact on the psychosocial well-being of affected couples [11, 12]. In this study, we found that 25.9% of infertile couples with at least one partner had anxiety, depression, or both. Of them, 18.7% of females and 13.7% of males were anxiety and/or depression disorders. A previous study showed that 40.2% had a psychiatric disorder among 112 women visiting the assisted reproduction clinic, including 23.2% with anxiety disorder, and 17.0% with depressive disorder [5]. Liu et al.’s study also found that the incidences of anxiety and depression on the first day of entering the IVF-ET cycle were 29.96% and 15.79% in women, and 20.65% and 13.77% in men among 247 infertile couples, respectively [15]. These studies demonstrated that anxiety and depression disorders were common in infertile couples. Consistent with Liu et al.’s study [15], most of them were mainly mild anxiety and depression in our study. It is generally considered that women are primarily blamed in cases of infertility, so women are more vulnerable to being affected by infertility than men [21, 22]. The prevalence of anxiety for females (13.5%) was significantly higher than that for males (8.7%) in this study. Liu et al.’s study also showed that the incidence of anxiety in women was higher compared to men [15]. Consequently, more attention should be paid to female partners.

Our study demonstrated that 52 females (22.3%) were comorbidity of anxiety and depression among 233 females who had anxiety and/or depression. Among 171 males who had anxiety and/or depression, 36 males (21.1%) were comorbidity of anxiety and depression. Anxiety and depression often co-existed in many people suffering from mental health conditions [23, 24]. The association of high levels of anxiety with COVID-19 complications and comorbid depression had been found amongst hospitalized COVID-19 patients [23]. 14% of patients with Parkinson had a comorbid depressive disorder with anxiety [24]. More importantly, we found that SAS and SDS scores were positively associated with their own SDS and SAS scores. Their own anxiety and depression were independent risk factors for depression and anxiety, respectively. Similar to our findings, anxiety was an independent risk factor for depression, and depression was a risk factor for anxiety in COVID-19 patients [25]. Collectively, these studies indicated that it could present interactions between anxiety and depression in infertile couples. Compared with either disorder alone, comorbid depression and anxiety could increase impairment [26, 27]. Therefore, to more effectively psychosocial intervention and support, anxiety, and depression should be intervened simultaneously under the condition of the comorbidity of anxiety and depression in infertile couples.

Another finding in our study was that female SAS and SDS scores were positively associated with male SAS and SDS scores. Partners’ anxiety or depression were risk factors for anxiety/depression in infertile couples. A previous study showed that men with anxious partners were vulnerable to having depressive and anxious symptoms [28]. There was a strong correlation between psychological stress and psychopathology not only within the subject but also between the male and female partners within the couple [28]. Based on these studies, infertile couples may benefit from the treatment of both partners. Besides the above risk factors, low education levels, no children, and female unemployment were also risk factors for female anxiety. Liu et al.’s study also showed that the incidence of anxiety in women was related to education level, and annual family income [15]. The prevalence of depression was higher among women with a family income ≤ 3000 CNY/month [16]. Oman‑Samani et al. showed that a higher education level was less likely to develop anxiety symptoms [29].

The SAS and SDS scores of infertility couples significantly declined during ART. Massarotti C et al.’s study also indicated that the levels of anxiety and general distress were significantly decreased and the quality of life was improved during IVF [30]. Since the first baby was born using IVF in 1978 [31], ART has great progress and is now widely accepted by the public. As of 2019, the total number of births achieved through ART likely exceeded 8 million globally [32]. ART can resolve their infertility and give hope to infertile couples. In addition, knowing other infertile couples in the waiting room helped them to feel less alone in their problems. Therefore, anxiety and depression could be relieved during the process of ART.

## Conclusions

Female partners were more vulnerable to having anxiety. Low education levels, no children, and female unemployment were risk factors for female anxiety. More importantly, there was the comorbidity of anxiety and depression in infertile couples, and the levels of anxiety were positively associated with the levels of depression. The anxiety/depression of their own or their partner were independent risk factors for the anxiety/depression of infertile couples. Therefore, to cope with these risk factors, anxiety and depression should be simultaneously counseled, and their partners also should be given supportive psychotherapy.

### Limitations and strengths

In our study, we enrolled 1247 infertile couples which was a larger sample size compared to the previous studies. To avoid selection bias, we consecutively recruited patients into the study. Data was collected from female and male partners and from three-time points including before and during ART. The levels of anxiety and depression and risk factors for anxiety and depression in infertile couples were comprehensively assessed. However, there were two limitations to our study. On the one hand, we recruited patients from a single center of reproductive medicine treatment in China. Due to cultural and economic differences, the prevalence of anxiety and depression in infertile couples could be inconsistent with the other regions of China and other countries. On the other hand, because we only screened the mental state of infertile couples but not the fertile couples in our center during the same period, the prevalence of anxiety and depression between infertile couples and fertile couples can’t be compared.

## Data Availability

The data used during the current study are available from the corresponding author on reasonable request.

## Abbreviations

ART: Assisted reproductive technology (ART)
OR: Odds ratio
SAS: Self-Rating Anxiety Scale
SDS: Self-Rating Depression Scale
ANOVA: Analysis of Variance
